# An Exploration of the Views and Perspectives of Australian Trans and Gender Diverse Individuals About Transvaginal Ultrasound

**DOI:** 10.1111/psrh.70056

**Published:** 2026-01-27

**Authors:** Caitlyn Wilke, Sav Zwickl, Jane Chalmers, Nayana Parange, Shae Maple, Sarah McMullen‐Roach

**Affiliations:** ^1^ School of Allied Health and Human Performance Adelaide University Adelaide Australia; ^2^ Trans Health Research Group, Department of Medicine The University of Melbourne Melbourne Australia

## Abstract

**Background:**

Transvaginal ultrasound (TVS) is used routinely in gynecological care in Australia to manage gynecological health concerns. Typically, TVS is well tolerated by patients, with low levels of discomfort reported. Trans and gender diverse people assigned female at birth may experience gender dysphoria or testosterone‐related anatomical changes, which could make such intimate examinations physically difficult or emotionally distressing. However, to date, no studies have considered the impact of gender identity on individuals' experiences of TVS. To fill this research gap, we explored the experiences of TVS among trans and gender diverse individuals assigned female at birth within Australia.

**Methods:**

We conducted semi‐structured interviews with trans and gender diverse individuals assigned female at birth who have experienced TVS in Australia. We analyzed all interviews in line with Braun and Clarke's reflexive thematic analysis.

**Results:**

Ten trans and gender diverse individuals aged between 18 and 50 years old participated in this study. From their interviews, we developed three overarching themes: (1) *It's a bit like being a detective*, (2) *So I could properly say*, “I don't want this done,” and (3) *I definitely felt like a novelty*. Participants described a range of positive and negative experiences with TVS, with issues related to cisnormativity in documentation, staff attitudes, and inadequate informed consent consistently highlighted.

**Conclusion:**

Trans and gender diverse people face challenges in accessing inclusive gynecological care in Australia. Our findings highlight a need for improved informed consent guidelines, better education and training for health professionals, and more inclusive clinic documentation to promote inclusive care.

## 1 Introduction

Trans and gender diverse people^1^ have a gender identity different from that typically expected of their sex assigned at birth.^2^ This includes a diverse range of identities and experiences, including, among others: trans men, trans women, non‐binary and fluid genders, and culturally‐specific gender identities, such as Aboriginal Brotherboys and Sistergirls [6, 7].

As a result of this incongruence between sex and gender, trans and gender diverse individuals may experience gender dysphoria [4, 7]. Gender dysphoria is a marked discomfort or distress that may occur in relation to the individual's body, particularly secondary sex characteristics, and/or how their gender is perceived by others [4]. To alleviate gender dysphoria, many trans and gender diverse individuals desire the use of gender‐affirming hormone therapy and/or gender‐affirming surgery to align their physical characteristics and gender identity [8, 9, 10].

In trans and gender diverse individuals assigned female at birth, testosterone hormone therapy typically results in a range of physical changes, such as increased body and facial hair growth, increased muscle mass, and a deepened voice [11]. Testosterone may also affect the genitalia and reproductive organs, leading to changes such as vulvovaginal atrophy, clitoral enlargement, endometrial and ovarian changes, and chronic pelvic pain [12, 13, 14, 15, 16].

As most individuals do not undergo gender‐affirming hysterectomy and genital surgeries, many trans and gender diverse individuals still require gynecological healthcare services such as a transvaginal ultrasound (TVS) [8]. These anatomical and hormonal changes can influence both the physical experience of the procedure and the emotional response to it [5].

Sonographers across Australia routinely use TVS to investigate and manage gynecological health concerns such as dysfunctional bleeding, endometriosis, and pelvic cancers [17, 18]. TVS involves inserting an ultrasound transducer into the vagina, which emits sound waves into the pelvic cavity to generate images of the cervix, uterus, and ovaries [17]. For cisgender women, TVS typically causes minimal discomfort, embarrassment, or pain [19, 20, 21, 22, 23]. However, to date, no studies have examined the tolerability of TVS in trans and gender diverse populations.

Research into similarly intimate procedures (e.g., cervical screening) shows that trans and gender diverse individuals assigned female at birth participate in these scans at lower rates (~38.9%) [24] than cisgender individuals (54%–60%) [25], and consequently report higher cervical cancer rates [24, 26, 27]. Several factors contribute to this lower participation, including insufficient patient education, physical pain and discomfort, and negative staff response to patients' gender identity [28, 29].

Additionally, the use of gendered terminology (e.g., “female reproductive organs” or “vagina”) when referencing anatomical areas during screenings increases discomfort in some [30]. These factors, combined with the necessity to undress and expose one's genitalia to strangers during screenings, may exacerbate gender dysphoria related to the genitals and/or reproductive organs [24].

Given that TVS requirements are similar to cervical screenings (exposed genitalia, insertion of a medical device into the vagina, and highly gendered language), it can be inferred that TVS may be avoided for similar reasons. While international literature highlights challenges in healthcare for transgender and gender diverse communities, there is limited evidence on experiences specific to the Australian context, particularly relating to TVS. Despite the widespread use of TVS, no research has explored its impact on trans and gender diverse people. Thus, our study aimed to capture the views and perceptions of trans and gender diverse individuals with TVS through the research question “What are the views and perceptions of trans and gender diverse individuals assigned female at birth about transvaginal ultrasound within Australia?”

## 2 Methods

### 2.1 Positionality Statement

Historically, trans and gender diverse people have been stigmatized and pathologized in research, resulting in misrepresentation, underrepresentation, and erasure within academic literature [31, 32]. Acknowledging this history, we approached this study with awareness of our own personal experiences and the need to minimize bias throughout data collection and analysis. To support this, we assembled a diverse research team that included both trans and cisgender people, as well as people of different ethnicities, socio‐economic backgrounds, education levels, and sexualities. Our team also included researchers with expertise in trans and gender diverse health, LGBTIQA+^3^ health research, sonography, and pelvic health physiotherapy.

### 2.2 Research Design

We employed a qualitative descriptive methodology for this study due to its ability to capture subjective data for direct participant quotation [34, 35]. This allowed insights into the experience of trans and gender diverse individuals with limited researcher inference, thereby increasing the credibility and trustworthiness of the study [36, 37].

We created and piloted an initial semi‐structured interview guide with one trans individual assigned female at birth who had undergone pelvic floor assessments, but not TVS. We then submitted this guide for community consultation with trans and gender diverse lived experience researchers from the Trans Health Research Group. This helped refine question appropriateness and response effectiveness without limiting the sample population. Table 1 contains an overview of the finalized semi‐structured interview schedule after this consultation.

**TABLE 1:** Semi‐structured interview schedule.

Area covered	Main questions asked
Introduction	Could you tell me more about yourself and what prompted you to participate in this research?
	2 What is your preferred terminology when it comes to discussions around this topic? (e.g., would you prefer we refer to TVS as just an internal ultrasound, etc.)
Prior to first TVS appointment	Before attending the appointment for your TVS, would you say you understood what the TVS procedure was and how it would be performed?
	2 Could you talk me through how you learnt more about this and any resources you may have used?
	3 How were you feeling about having the procedure? Could you talk me through any emotions related to it.
	4 Could you talk me through how you prepared for the procedure?
	5 Could you talk me through how you went choosing where to have your transvaginal ultrasound?
	6 If you could make any recommendations for the preparation stage, to either doctors or other members of your community, what would you recommend?
Experience during TVS	Once you were aware of the process and knew you had to have it, how did you go about booking your appointment?
	2 When you arrived at your appointment and you had to check in with the administration staff did anything in particular stand out to you? Could you talk me through that, and how it made you feel.
	3 Once you got there and were in the waiting room, how were you feeling?
	4 What was your experience with the procedure itself and the person who did the scan?
	5 Did you find that you had a chance to express your preferred pronouns at any point from arrival to during the procedure?
	6 Was there anything about the TVS process or anything the health practitioner did that made you feel more comfortable or uncomfortable? How was this done?
	7 Do you think the way your gender related appearance/the way your gender was perceived affected this process at all, for better or for worse?
Experience after TVS	Hypothetically if you were to give any feedback to the organization or health practitioner what would it be?
	2 Would you recommend this imaging service to other members of your community? Why or why not?
	3 Has your experience with TVS changed how you interact with health care professions? Why or why not?
Conclusion	Is there anything further about your experience with TVS that you would like to share or discuss that I have not brought up yet or that you would like to revisit?

### 2.3 Participant Sampling and Recruitment

We recruited participants using a combination of snowball and purposive sampling, with the following inclusion criteria: aged 18 or over; assigned female at birth (sex); identified as a gender other than female or woman (gender); had undergone at least one TVS at an Australian practice; resided in Australia; and had access to a reliable internet connection for a video interview. We excluded participants if they had limited English proficiency or impaired ability to describe their experience in English.

The hard‐to‐reach aspect of trans and gender diverse populations necessitated recruitment through digital flyers through Australian LGBTIQA+ organizations, such as ShineSA to better reach the target population [38]. We also recruited through our professional and personal networks.

Potential participants registered their interest through an online screening survey hosted on Qualtrics (v. March 2024, Qualtrics, Provo). This screening survey was used to assess eligibility, including questions related to participants' age, sex assigned at birth, gender identity, experience of TVS within Australia and time since their last TVS. The primary researcher (CW, cisgender woman, honors student) then contacted potential participants to confirm eligibility and schedule interviews.

**FIGURE 1:**
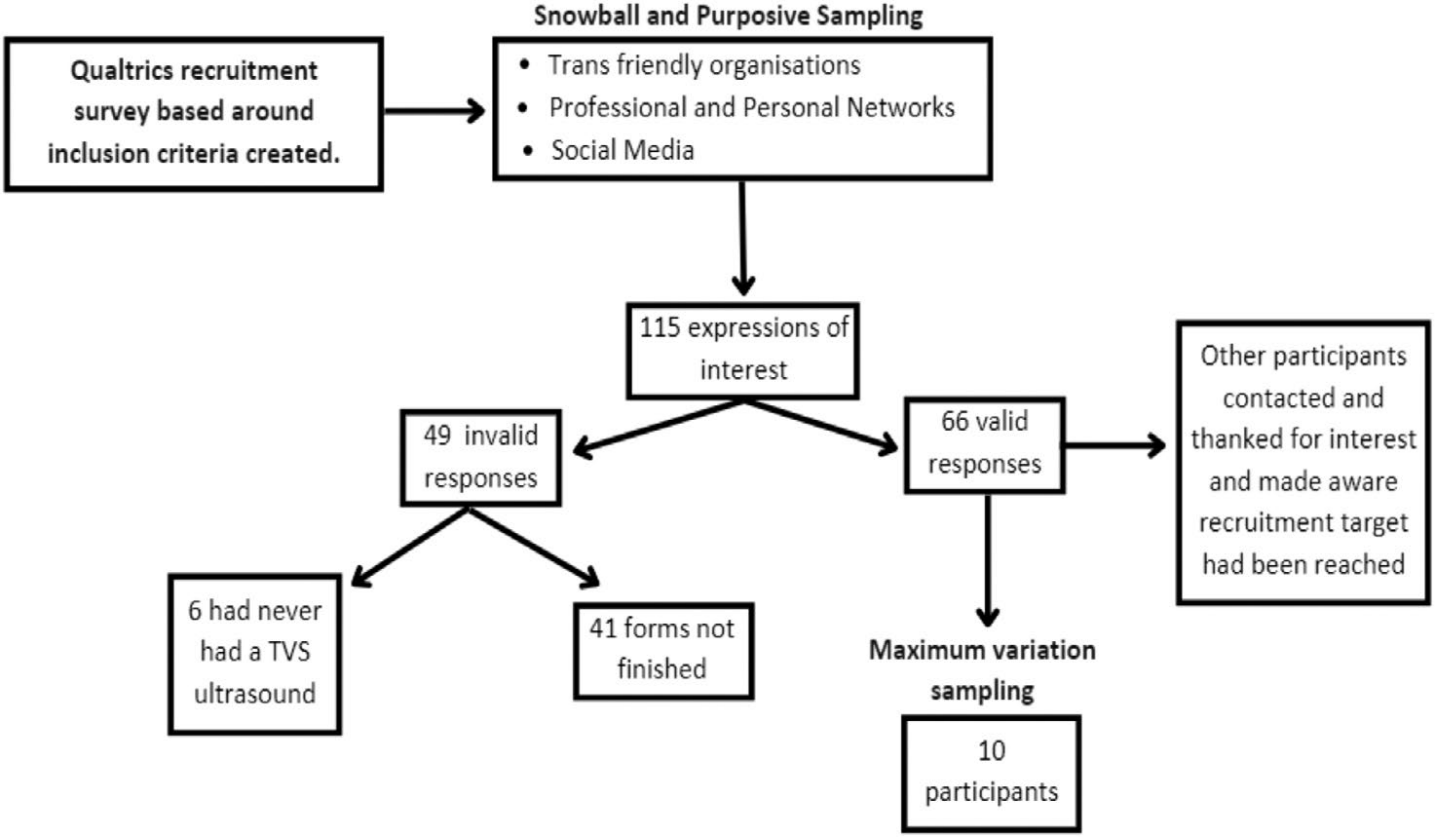
Flowchart of the sampling process used during participant recruitment.

To capture differences in experiences across demographics and locations, and increase transferability of the collected data, we conducted maximum variation sampling on screening survey responses. We aimed to diversify participants by location, age, and gender identity, as shown in Figure 1.

Given our study's specific inclusion criteria, we limited the sample size to 10 participants due to high information power. Information power states that the higher the relevance the information has to the study, the lower the number of participants needed so that significant reflection and analysis of each transcript could occur [39].

### 2.4 Data Collection

To familiarize participants with the question areas, we provided them with interview guides prior to interviews. The primary researcher conducted semi‐structured interviews using Microsoft Teams (v. 24193.1805.3040.8975, Microsoft, USA) or Zoom (v. 6.0.11, Zoom Video Communications, USA) from January 2024–April 2024 with audio and visual data being captured. Interviews lasted 30–120 min, with a mean length of 49 min. Upon interview completion, the primary researcher transcribed all video and audio data verbatim before destroying or deleting the original files. These transcripts were then uploaded to a secure, password‐protected database at Adelaide University, where they will be stored for five years in accordance with the *Australian Code for Responsible Conduct of Research* [40], after which they will be permanently destroyed.

### 2.5 Data Analysis

We conducted data analysis using Braun and Clarkes' six phases of reflexive thematic analysis [41]. The primary researcher manually transcribed all interviews verbatim. Then, to increase the dependability and confirmability of our findings, five researchers independently and manually coded in duplicate an initial set of transcripts (*n* = 5) to ensure limited researcher inference.

We then compared the coded transcripts and confirmed results through collaborative discussion to identify common patterns and generate initial themes. We used an inductive approach, using the semantic meanings generated from the data to lead theme formation in place of predefining themes.

After these collaborative sessions, the primary researcher refined initial themes against raw data, before reconvening with the research team to finalize and name themes. After the report was written, the results were returned to participants for member checking and selection of a pseudonym.

Throughout all stages of the study, the primary researcher maintained a reflexive journal to monitor their subjectivity during data collection and analysis. The authorship team met regularly for debriefing, where members shared and, when necessary, challenged the rationale behind decisions made during interviews and data analysis. These practices aimed to reduce the impact of individual subjectivity on the finalized themes.

### 2.6 Ethical Considerations

We obtained ethical approval from the University of South Australia's ethics board [ID: 205389], as well as thorough ACON, one of Australia's largest LGBTQ support organizations [RERC Reference Number: 202335], prior to study commencement. Participation in the study was voluntary, and all participants provided written informed consent. They could withdraw from the study at any time prior to their interview. All participants chose their own pseudonyms. To protect anonymity, individual details such as age, gender identity, and location have not been reported.

Two participants were known to the primary researcher through personal and professional networks used during recruitment.

As this study was unfunded and completed as part of honors research, we were unable to remunerate participants.

Generative AI was not used during the preparation of this manuscript, nor was it used in the generation or analysis of any data presented.

This research is reported per the consolidate criteria for reporting qualitative research (COREQ) checklist (Supporting Information 1) [42].

## 3 Results

We interviewed 10 participants between December 2023–April 2024. All participants were assigned female at birth, and one participant reported an innate variation of sex characteristics (often termed intersex) (Table 2).

**TABLE 2:** Participant demographics for included participants (*n* = 10).

Self‐identified gender	(*n*)
Trans man	1
Male	1
Male/Man	1
Transmasculine man	2
Non‐Binary/masculine	1
Non‐binary/trans masculine	1
Non‐Binary	2
Butch female	1
Pronouns used	
He/him	3
They/he	2
He/they	2
They/them	2
She/him	1
Age range	
18–25	5
30–34	1
35–39	2
40–44	1
50–54	1
State	
South Australia	5
New South Wales	2
Australian Capital Territory	1
Victoria	1
Queensland	1

### 3.1 Themes

We developed three main themes during data analysis, each with 2–3 subthemes, that explored participants' experiences with TVS (Table 3). These themes captured experiences with consent, service accessibility, general TVS education, and the education of clinicians around trans and gender‐diverse health.

#### 3.1.1 Theme 1: It's a Bit Like Being a Detective… (Eike, Non‐Binary Person)

This theme describes how participants ascertained how inclusive, and thus safe, a healthcare service was. In assessing for inclusion, participants identified clues such as inclusive symbols and signposting, and conversely signs of exclusion or discrimination.

##### 3.1.1.1 Basic Stuff That… Lets Me Know It's a … Safe Place (Zel, Non‐Binary Person)

Once participants were referred for TVS, they described the process of determining which practice they would attend. Participants shared that, when evaluating how inclusive and safe a practice felt, they paid attention to public statements about inclusivity as well as the language used on the clinic's website and in its documents.

Gendered language (e.g., “woman” (Juno, butch female)) was seen as non‐inclusive and communicated a lack of recognition “that it's not just cisgender females” (Eike, non‐binary person) getting TVS. Participants described “anatomical” or “procedural or medical” (Eike, non‐binary person) terms as more trans and gender diverse inclusive.

Intake forms were raised as not reflective of trans or gender diverse experiences, namely through the option of only binary gender choices, a lack of separate sex and gender options, and feminine‐specific honorifics. This led to some participants feeling the need to “Make [their] own boxes” (Lee, non‐binary/masculine) to accurately portray themselves.

I drew my own […] I did a separate gender and sex thing. And obviously, I understand the systems aren't meant to support [gender diversity]. (Lee, non‐binary/masculine)

Whilst all participants expressed a preference for the inclusion of pronouns and affirmed names^4^ in intake forms, it was asserted that this was only useful if used by staff.

I think [pronouns] should be on […] whatever paperwork staff involved in the patient's care take […] to ensure that people are being referred to respectfully. There are many trans people whose legal documentation or Medicare doesn't line up with their actual name or gender, so that can be very distressing. (Robbie, man)

[Reception staff] see the ‘F’ [on legal documents] […] they like, you know, use ‘she/her’. They make those assumptions. (Rex, non‐binary transmasculine)

Some participants described assessing the physical environment to determine inclusivity. Physical symbols such as pronoun badges, rainbow flags, or “Rainbow Tick” certification^5^ (Eike, non‐binary person) were raised as explicit symbols that communicated “a comfortable, safe place” (Zel, non‐binary person).

Less explicit signs included the inclusion of gender‐neutral bathrooms. Sebastian reflected on how attending a clinic that only had a women's bathroom led to him worrying about being “harassed” as “it didn't need to be labelled as a woman's toilet […] in a private clinic”.

##### 3.1.1.2 It Would Make Sense for Access and Inclusion to Grow Together (Aaron, Trans Man)

Whilst many participants indicated a preference for an inclusive practice, their choice of location was superseded by considerations of affordability, convenience, and availability.

They [referrers] specifically wanted me to go to a specific, non‐bulk billing sonography clinic that specializes in pelvic scans, but like, I can't afford it. (Aaron, Trans man)

Conversely, some participants reflected that attending a practice with staff who were trans and gender diverse inclusive was more important than cost. Marcus decided to go to a hospital because he felt that the staff at a private practice^6^ “may not have been used to dealing with people with beards getting TVS.”

**TABLE 3:** Overview of themes and subthemes.

Main themes	Subthemes	Theme summary
1. “It's a bit like being a detective”	1.1: “Basic stuff that… lets me know it's a … safe place”	This theme describes how participants ascertained how inclusive, and thus safe, a healthcare service was. In assessing for inclusion, participants identified clues such as inclusive symbols and signposting, and conversely signs of exclusion or discrimination.
	1.2: “It would make sense for access and inclusion to grow together”	
2. “So I could properly say,’ I don't want this done’”	2.1: “I didn't know what it was or that I was getting it”	This theme describes the role that education around TVS plays on participants' feelings of empowerment and control during and before a scan, and the impact this had on participants perceived consensual practice from staff.
	2.2: “I'm not being told, ‘you have time to consider this’”	
	2.3: “It was a long time before I realized I could say no”	
3. “I definitely felt like a novelty”	3.1: “I tend to assume that people don't have a clue about trans people”	This theme describes aspects of inclusive and non‐inclusive care received by participants, highlighting how provider education and attitudes can impact feelings of safety and inclusion. This also incorporates recommendations from participants on how sonographers can provide inclusive care.
	3.2: “Not performing gender correctly”	
	3.3: “It didn't take any longer […] being inclusive”	

Additional barriers and accessibility needs of participants further impacted location considerations—such as the availability of patient handling and transfer equipment. This “double marginalization” (Zel, non‐binary person) was important to highlight as “the amount of trans people and the crossover between chronic pain and disability or neurodivergence[…] is huge” (Aaron, Trans man).

This highlights that intersectional identities, experiences, and needs impact TVS access.

#### 3.1.2 Theme 2: So I Could Properly Say, ‘I Don't Want This Done’ (Lee, Non‐Binary/Masculine)

This theme describes the role that education around TVS plays on participants' feelings of empowerment and control during a scan.

##### 3.1.2.1 I Didn't Know What It Was or That I Was Getting It” (Lee, Non‐Binary/Masculine)

Participants reported inconsistent information provided before their first TVS. Whilst all participants indicated that they were well informed about “when to drink water […] having a full bladder” (Robbie, man), there was a varied understanding that the scan involved inserting a transducer into the vagina.

So, I just rocked up then the technician was the one who said, ‘Oh now we're going to do the internal part of it’. And I'm like, ‘Excuse me?’ (Eike, non‐binary person)

Participants described inadequate descriptions of TVS at the time of their referral. This resulted in participants feeling disempowered in making informed decisions about proceeding with TVS.

I did not know that when he [referrer] said, ‘internal’, he meant it will go into your vagina. If I had, I would have been like, ‘Oh no, I don't feel comfortable with that’. (Zel, non‐binary person)

[…]being given proper solid information of what I was going in for, what I was expecting […] so I could properly say, ‘I do not want this done[…] this will make me cry’. (Lee, non‐binary/masculine)

Lack of information also eroded participants' trust in their referrer's knowledge of the procedure, as well as their referrer's clinical judgment.

Conversely, other participants described receiving education from referrers that “described the process” (Rex, non‐binary transmasculine). For example, Sebastian received information in “various formats” that included printed information, YouTube videos, and additional chats with his referrer.

In place of education from referrers, some participants turned to members of the trans and gender diverse community. Participants indicated that they found community perspectives offered unique insights into “what others' experiences were like” (Aaron, Trans man) and recommendations on trans and gender diverse inclusive providers.

Participants who accessed information from community sources were satisfied with their insights, and described how “they knew what to expect and why it was done that way” (Marcus, male/man). This generally led to a more positive experience compared to those who received information solely from their referrer.

##### 3.1.2.2 I'm Not Being Told, ‘You Have Time to Consider This’ (Zel, Non‐Binary Person)

Participants who lacked adequate information before TVS typically learned about the internal component from sonographers after the external scan ended. This left some participants feeling they lacked adequate time to consider whether they wanted to proceed with TVS.

I think providing the information with ample time before the procedure rather than like the day of, when you're already sort of heightened [emotionally]. (Sebastian, transmasculine man)

This was raised as a unique concern for TVS examinations, as the diagnostic nature of these scans left some participants feeling anxious about results. Consequently, some participants recalled agreeing to proceed with TVS due to health concerns without fully understanding what they were consenting to.

I was more focused on the diagnostic outcome, like what's going on, what's causing the pain, and needing to do something about this pain. (Eike, non‐binary person)

This highlights that the timeliness of information provision is important to participants. Providing information about the internal component of a TVS during the examination, when participants may already be anxious, could lead to agreement without full comprehension.

##### 3.1.2.3 It Was a Long Time Before I Realized That I Can Actually Say, ‘No’ (Eike, Non‐Binary Person)

All participants described how important informed consent throughout TVS was in relation to feelings of control and the ability to relax.

Some participants described that sonographers who used continuous dynamic consent led to them feeling in control during the TVS. This consent process encompassed “talking it through step by step” (Sebastian, transmasculine man), verifying consent to begin the internal portion and “checking in” (Marcus, male/man) with participants either verbally or non‐verbally—such as offering participants a hand to squeeze to terminate TVS.

Some participants described preferring to receive information in a stepwise manner throughout the procedure, as well as receiving warnings from the sonographer before inserting or moving the transducer.

[…] being talked through it, felt more like I was actively involved rather than […] a passive participant that was getting something uncomfortable done to me. (Sebastian, transmasculine man)

Checking in was defined by participants as confirming with them, “are you happy to continue?” (Eike, non‐binary person) before the internal component. Having the opportunity to give explicit ongoing verbal consent was highlighted as being important in enabling participants to get “ready or likewise go, ‘Actually I'm not in the headspace for that’.” (Eike, non‐binary person). This was identified as foundational in empowering participants to say no to TVS as it explicitly highlighted an “opt out” (Sebastian, transmasculine man).

They're [sonographer] like, ‘So now we're going to do, like, the internal ultrasound, if that's something you're comfortable with?’[…] it was very consensual. (Juno, butch female)

Some participants raised that this dynamic consent and stepwise communication of information increased comfort and lessened feelings of gender dysphoria.

Conversely, participants who had rescinded their consent reported experiences where sonographers applied verbal pressure to complete the scan.

I have said, ‘No’ before because I was incredibly dysphoric[…] I do not want anybody touching down there[…] and [the sonographer] was done with the pelvic [scan] and she said, ‘We are gonna do the TVS now’[…] not like, ‘Are you okay if we move onto the TVS?’ And I said, ‘No, I'm not comfortable’ and she [said], ‘You need it’. (Percy, transmasculine man)

The resultant emotional distress caused an inability to relax, leading to physical pain and incomplete scans, with one participant reporting that this experience made them hesitant for future scans.

Being able to give and rescind consent throughout the scan was identified as paramount to participants' comfort and sense of autonomy.

#### 3.1.3 Theme 3: I Definitely Felt Like a Novelty (Sebastian, Transmasculine Man)

This theme explores the factors of care participants raised as being inclusive or non‐inclusive during TVS. It also incorporates participant recommendations on how to improve care for trans and gender diverse people undergoing TVS.

##### 3.1.3.1 I Tend to Just Assume That People Don't Have a Clue About Trans People (Robbie, Man)

Some participants reported that sonographers lacked education on trans and gender diverse healthcare and inclusive practice. Participants described experiences that reflected a lack of sonographer knowledge and/or appropriate approaches to the genital and internal organ changes that can result from testosterone use. For example, Percy shared how one sonographer asked him when he got his “vagina created,” indicating a lack of understanding of the difference between trans and gender diverse people assigned female at birth and those assigned male at birth. Marcus directly contrasted two of his TVS scans, one before and one after commencing testosterone. Following testosterone commencement, he described that the sonographer found it harder to “Get a good look” and attributed this to not having drunk enough water, without consideration of the possibility that it was due to vaginal atrophy from testosterone.

Participants also described that sonographers typically placed the onus of trans and gender diverse education on patients themselves, often through questions irrelevant to care. Percy reported that when sonographers were not “ignoring anything” he said about being trans, they were “hounding” him with questions such as “How does the surgery work?” In response to their flat chest, Eike was asked, “Have you had breast cancer[…] or have you had top surgery?” These questions were seen as intrusive and impacted confidence in sonographers' provision of care.

##### 3.1.3.2 Not Performing Gender Correctly (Zel, Non‐Binary Person)

Participants reported that staff attitudes varied on the basis of their gender presentations. Participants who described being perceived as feminine in appearance summarized “frustrating” experiences of being assumed as “female” with no consideration of other gender identities. Percy described how he was seen as “not masculine enough” to be trans and was treated as a “quirky girl” when he communicated that he was a transmasculine man. Zel reflected that when they dressed in a way that was more feminine and in line with female gender norms, they were treated “more seriously” than when they dressed androgynously.

Some participants, who reported being perceived as masculine at the time of their appointment, reported no issues with staff attitudes toward their appearance. For example, Sebastian described how when staff “did not bat an eyelid” over him being trans, this removed the “burden” from him of feeling, “I need to explain[…]or justify why I was in the room.” However, Marcus described how sonographers seemed “standoffish” and less willing to answer questions, an experience which he perceived as discriminatory.

##### 3.1.3.3 It Didn't Take Any Longer […] Being Inclusive (Percy, Transmasculine Man)

Participants described a range of experiences with sonographers that they considered indicative of inclusive care. These included instances where the sonographers employed appropriate bedside manner, directly addressed patients' concerns, used privacy devices, offered participants the ability to insert the transducer themselves, and were conscious of the language used.

Some participants noted that the way sonographers exhibited awareness of the importance of gender inclusive and affirming language was through the use of patients' affirmed names, procedural rather than gendered terminology, and the terms used in general conversation. When gender inclusive and affirming language was not used, this led to “misgendering” (Eike, non‐binary person) and “deadnaming” (Percy).

Whilst all participants highlighted that anatomical or medical language (e.g., external genitals) was more inclusive than colloquial gendered language (e.g., vulva), only one participant reported being asked about their terminology preference

So, they're [sonographers] like, ‘Is it OK if we say like vagina?’ and like I said, ‘Totally fine, like, because I've got that’. (Sebastian, transmasculine man)

Some participants recalled sonographers' referencing shared female experiences, through use of terms such as “us girls” (Eike, non‐binary person) or “isn't it hard being a woman?” (Percy, transmasculine man). Such language communicated gender assumptions that did not align with the participants' actual gender.

Participants highlighted that when gender inclusive and affirming language was used, they relaxed more, making the scan more physically comfortable.

It was literally just her [sonographers] choices of wording[…] like, being inclusive and respectful[…] I was also a lot more comfortable and it didn't hurt as much because guess what? I was relaxed. (Percy, transmasculine man)

The use of gender inclusive and affirming language in conjunction with typical sonography care resulted in more positive experiences for participants.

## 4 Discussion

This study uniquely contributes Vto the literature by capturing the views and perceptions of trans and gender diverse individuals assigned female at birth about TVS. Participants reported more positive experiences when they encountered inclusive, consensual, and affirmative care, which facilitated feelings of inclusion, safety, and control. These findings align with broader imaging research, where transgender and gender diverse patients have reported emotional discomfort, misgendering, and negative imaging encounters during ultrasound procedures as the highest contributor of unexpected discomfort [45]. The key factors that impacted this were the language used in clinic environments and documentation, perceptions of informed consent throughout TVS, and the knowledge of referring providers and medical imaging staff around trans and gender diverse inclusive and affirmative care.

Findings from this study were consistent with broader healthcare research that feelings of inclusion and safety were not being met for trans and gender diverse individuals [46, 47, 48]. Participants' initial perceptions of inclusion, and subsequently safety, were impacted by clinical documentation language, as well as trans and gender diverse supportive signs or symbols. While these findings emphasize the role of visible signs and symbols of inclusion, broader medical imaging literature similarly demonstrates that environmental and healthcare practices shape patients' perceptions of safety and autonomy [49]. A review by Hammond and Lockwood [50] found these areas were common in assessing inclusivity within healthcare by trans and gender diverse individuals. Similarly, Grimstad et al. [45] found that many imaging environments lacked visible affirming cues such as LGBTQ+ signage or gender‐neutral facilities, contributing to feelings of discomfort and alienation among trans and gender diverse patients.

Intake forms that only contained binary gender options and lacked pronouns or affirmed name sections communicated exclusion of trans and gender diverse individuals through cisnormativity [51, 52]. Since many trans and gender diverse people have not or are unable to legally change their name and/or gender marker [53, 54, 55], intake forms that don't allow patients to include a name and gender different to their legal name and gender increase the likelihood of deadnaming and misgendering [56, 57]. It is well‐established within the literature that deadnaming and misgendering can be a severe source of distress and contribute to healthcare avoidance [31, 48, 58, 59, 60]. As TVS is used for diagnostic purposes, avoidance due to non‐inclusive environments or documentation could contribute further to poor health outcomes within this population.

The need for improved informed consent guidelines for TVS practice was also highlighted in our study, and is supported by the literature [61]. Informed consent begins at referral, with education provided to participants enabling them to make informed decisions about their care. As shown in our study and in the broader literature [22, 62, 63, 64], referrers aren't adequately educating patients before intimate examinations. Patients are then attending appointments they do not fully understand, which increases the risk of harm [64] and unethical practice, as consent is incorrectly assumed by staff [60, 65].

Informed consent is important from the referral through to the scan. As raised in the Australasian Sonographer Association guidelines, a power imbalance exists between clinicians and patients in intimate examinations due to the patients' vulnerable state [66]. By providing patients with information on the internal portion of TVS only after the external scan has finished, patients are less likely to voice concerns [67], and are more likely to choose to undergo TVS as they see it necessary for care [22]. In our study, this led to direct disempowerment in participants' abilities to make informed decisions, with resultant emotional and physical discomfort.

Participants in this study indicated a preference for sonography actions that fostered feelings of control—namely, dynamic consent, checking in and being provided with direct ways to stop the scan. Consistent with research by McDowell et al. [67] and Reiesner et al. [68] on the use of vaginal swabs in trans and gender diverse patients, participants felt in control and reported fewer occurrences of gender dysphoria when these actions were undertaken. As reported by Carroll et al. [29], gender dysphoria may occur in intimate examinations due to the physical contact necessary for the examination and association with body parts that may cause gender‐related distress. Gender dysphoria is well‐documented as a cause of healthcare avoidance [69, 70, 71] and could pose further challenges to TVS for trans and gender diverse individuals. Thus, clearer and more accessible informed consent guidelines should be established for TVS. These should be provided to patients as soon as possible and offer accurate explanations in multiple formats to enhance accessibility and allow ample time to understand and prepare for their first TVS appointment. Sonographers should also further improve communication around withdrawal of consent and how patients can communicate this.

Alongside consensual practice from sonographers, participants emphasized how sonographer education impacted their trust in the sonographer's ability to provide respectful or competent care. Participants indicated that sonographers lacked training in gender‐affirming care, as well as an understanding of anatomical differences in trans and gender diverse populations. Pratt‐Chapman et al. [71] found that healthcare professionals reported a lack of education around the needs of trans and gender diverse and intersex patients, resulting in low confidence and overall professional discomfort in providing care to these populations. Previous research suggests that a lack of LGBTQ+ awareness can lead to increased prejudice and discrimination from healthcare professionals, which can contribute to patient avoidance of healthcare [58, 72]. Given the well‐documented low inclusion of LGBTQIA+ education within healthcare curricula worldwide [73, 74, 75, 76, 77, 78], it is likely that similar training gaps exist within medical imaging practice, where limited guidance contributes to variability in communication, consent, and patient autonomy during imaging examinations [49]. This poses a risk for increased misdiagnosis rates, as disease presentation after testosterone commencement may vary from typical presentations [13, 76, 79]. Hence, this study further adds to the literature supporting the need for inclusion of trans and gender diverse specific education into sonography curricula. Inclusions of LGBT education in health provider curricula have been shown to increase providers' self‐confidence in providing care to trans and gender diverse individuals [80, 81], as well as improve culturally competent care more broadly [82, 83].

## 5 Strengths and Limitations

Reflexivity, coding in duplicate, and team‐based analysis for theme formation mitigated the risk of bias and increased the dependability and credibility of this study. Confirmability was achieved through member checking and the use of direct participant quotations [84].

Two participants in this study were known to and interviewed by the primary researcher. While this did not constitute a conflict of interest, it may have introduced bias into data collection or interpretation. To address this in part, coding of these interviews was done in duplicate [37].

Due to the voluntary nature of participation and the small sample size, a potential recruitment bias may be evident in our sample. Only participants who were able to access the internet and were willing to share their TVS experience were captured. This may have added a recruitment bias in that it is possible that older adults, lower socio‐economically impacted individuals, or those with significant gender dysphoria may have been less likely to participate and thus not have their perspectives captured. Future research should explore safe, trauma‐informed ways to include these voices.

Some participants had multiple scans, which could have influenced familiarity with the procedure and thus the results. Recall bias may have also impacted the data collected due to a lack of standardization on the time length from a participant's first TVS instead of their most recent TVS [85]. Further research should consider cultural, age, or educational impacts on trans and gender diverse experiences of TVS.

Additionally, further research should also consider if there is a difference in experiences between services provided by public and private health services within Australia. Whilst this was briefly mentioned in the study results, it was not collected during screening or interviews, and as such, the impact of public/private sector services was not within the scope of this study.

## 6 Conclusion

This paper uniquely contributes to sonographic practice by highlighting a range of positive and negative experiences with TVS, with consistent issues in practice regarding cisnormativity in documentation and staff attitudes, as well as a lack of informed consent considerations. These findings highlight the need for improved guidelines around informed consent practices within TVS, as well as health professional education around trans and gender diverse patients.

## Author Contributions

**Caitlyn Wilke:** conceptualization [lead], design of interview guide [lead], data collection [lead], formal analysis [lead], methodology [lead], project management [lead], visualization [lead], writing – original draft [lead], writing – review and editing [lead]. **Sav Zwickl:** formal analysis [supporting], methodology [supporting], supervision [supporting], writing – review and editing [supporting]. **Jane Chalmers:** conceptualization [supporting], methodology [supporting], investigation [supporting], formal analysis [supporting], supervision [supporting], writing – review and editing [supporting]. **Nayana Parange:** formal analysis [supporting], visualization [supporting], writing – review and editing [supporting]. **Shae Maple:** conceptualization [supporting], methodology [supporting], formal analysis [supporting], investigation [supporting], supervision [supporting], writing – review and editing [supporting]. **Sarah McMullen‐Roach:** conceptualization [supporting], formal analysis [supporting], investigation [supporting], methodology [supporting], supervision [lead], writing – review and editing [supporting].

## Funding

The authors have nothing to report.

## Ethics Statement

This study was approved by the Human Research Ethics Committee of the University of Adelaide (approval number: 205389) and ACON Research Committee (RERC 202335). All procedures were conducted in accordance with the National Statement on Ethical Conduct in Human Research.

## Consent

Written informed consent was obtained from all participants prior to participation. Participants were informed of the voluntary nature of the study and their right to withdraw at any time without consequence.

## Conflicts of Interest

The authors declare no conflicts of interest.

## Supporting information


**Data S1:** psrh70056‐sup‐0001‐supinfo.docx.

## Data Availability

Due to the sensitive and identifiable nature of the qualitative data generated and analyzed during this study, data are not publicly available. Reasonable requests for access to de‐identified data may be considered by the corresponding author, subject to ethical approval and participant consent.

## Endnotes

^1^
At a community‐level, there is no widely agreed‐upon term to describe people with a gender different from the gender assigned to them at birth. In this work, we use ‘trans and gender diverse’ as a community‐level term and avoid use of acronyms, as they can lead to a diminishing of identities for marginalized groups, and be seen as reductive and objectifying, in line with recommendations from a recent Australian terminology‐focused study and by the editorial team of the *International Journal of Transgender Health* [1, 2]. Trans is used rather than transgender here, as a term more inclusive of individuals who consider themselves transsexual but not transgender [3].

^2^
Assigned sex at birth refers to the attribution first made on a birth record where individuals are typically categorized as being either male or female, based primarily on visual inspection of the genitalia. The authors have chosen to use ‘assigned female at birth’ based on guidelines from the Trans Health Research Group, The University of Melbourne [4]. Another common term is ‘recorded female at birth’ [3, 5].

^3^
The term LGBTIQA+ refers to lesbian, gay, bisexual, transgender, intersex, queer, asexual people, or people otherwise diverse in gender, sexual orientation and/or innate variations of sex characteristics. There are a number of different acronyms used to refer to these populations, based on the context and the specific population being referenced. When referring to the broader population this paper uses LGBTIQA+ in line with Australian Institute of Family Studies [33]. Throughout this paper various abbreviations including LGBTQI, LGBTQA+ and LGBTIQA+ are used, to acknowledge what was used in the referenced research.

^4^
Affirmed name is also known as a chosen or preferred name, and is the name an individual goes by after affirming their gender/coming out [1]. In this work we use affirmed name over preferred name, as preferred implies a preference on use of the name which can undermine the importance of using someone's correct name.

^5^
Rainbow Tick refers to an Australian quality framework that is used by health and human service organizations to show that they are safe, inclusive and affirming services and employers for the LGBTIQ community. Accreditation for this is provided by the Quality Innovation Performance and Australian Council on Healthcare standards [43].

^6^
In Australia a dual public‐private healthcare system is used, with public services provided by state and territory governments, and funding provided by the Australian Government. Additionally, there are private medical imaging services, which are owned and managed by private organizations that set their own providers fees, typically leading to an out‐of‐pocket cost for patients [44].
